# Thermochemistry of cation disordered Li ion battery cathode materials, 
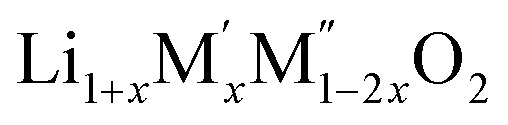
 (M′ = Nb and Ta, M′′ = Mn and Fe)†

**DOI:** 10.1039/c9ra09759g

**Published:** 2020-02-12

**Authors:** Tamilarasan Subramani, Alexandra Navrotsky

**Affiliations:** Peter A. Rock Thermochemistry Laboratory, NEAT ORU, University of California Davis CA 95616 USA; School of Molecular Sciences, Center for Materials of the Universe, Arizona State University Tempe AZ 85287 USA alexnav@asu.edu

## Abstract

High temperature oxide melt solution calorimetry studies on 
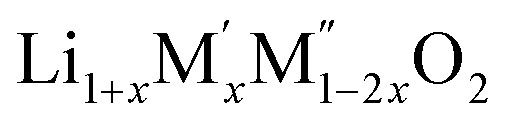
 (M′ = Nb^5+^, M′′ = Mn^3+^ and Fe^3+^ and *x* = 0.20, 0.30 and 0.40) oxides and a new family of Ta containing Li excess disordered cathode materials, 
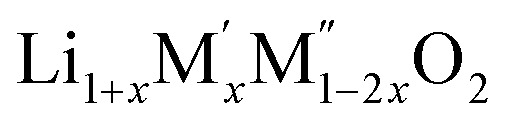
 (M′ = Ta^5+^, M′′ = Fe^3+^ and *x* = 0.20, 0.30 and 0.40), synthesized by a rapid quenching method, are reported in this study. The enthalpies of formation determined from high temperature calorimetry studies reveal that the stability of compounds increases with the increasing Li content per formula unit. The reaction between more basic Li_2_O and acidic transition metal oxides results in the more negative enthalpies of formation for these compounds. The work reveals that the formation enthalpy term 
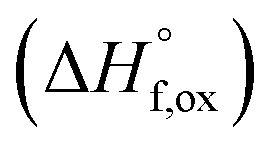
 plays a more important role in the stabilization of such disordered Li ion materials at room temperature whereas configurational entropy along with lattice entropy (vibrational and magnetic) contributes to the stabilization at high temperature from which the samples are quenched.

## Abstract 2

Enthalpies of formation from oxides (Δ*H*_f,ox_) of novel disordered Li_1+*x*_Ta_*x*_Fe_1−2*x*_O_2_ and reported  (M′′ = Mn^3+^ and Fe^3+^).

## Graphical abstract

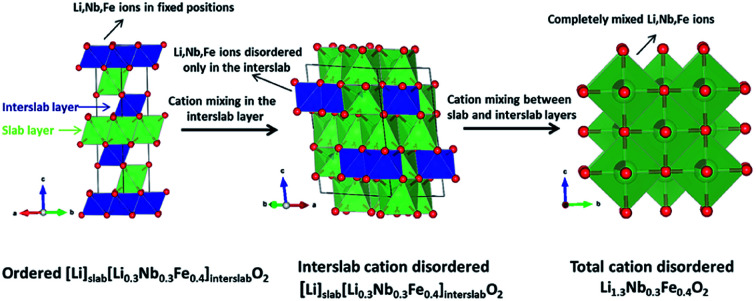

## Introduction

Lithium containing oxides are investigated extensively for application as cathode materials for lithium ion batteries (LIB). Such research is mostly focused on oxides with highly ordered structures including layered rock salt phases containing lithium and transition metals (M) such as LiMO_2_ and Li_2_MO_3_ and ordered spinels such as LiMn_2_O_4_ due to their high performance.^1–3^ Research on cation disordered materials is more limited as the community focused only on ordered materials.^4–6^

A lithium rich cation disordered material, Li_1.211_Mo_0.467_Cr_0.3_O_2_, has been shown to display a high discharge capacity and high energy density, suggesting that excess Li containing cation disordered materials could be a new class of cathode materials for LIB.^6^ The hopping of Li ions from one octahedral site to neighbouring octahedra *via* a tetrahedral intermediate site, similar to the pathway proposed in the ordered oxides is suggested as the diffusion pathway for Li ion migration in the disordered oxides.^6,7^ The difference in the Li diffusion in the ordered and disordered Li oxides arises in the intermediate state. In the intermediate state, diffusion of Li ions in the ordered Li oxides proceeds *via* channels that are surrounded by both Li and transition metal ions whereas in the disordered Li oxides, the diffusion occurs through channels which are completely surrounded by Li ions.^6,7^ Disordered oxides are Li-excess oxides compared to stoichiometric LiMO_2_. The excess Li helps in the diffusion by forming more Li only surrounded channels in disordered oxides. Many other cation disordered oxides have also been shown to exhibit high discharge capacities. There are many Li_3_NbO_4_ based materials (Li_3_NbO_4_)_*x*_–(M^2+^O)_1−*x*_ and (Li_3_NbO_4_)_*x*_–(LiM^3+^O_2_)_1−*x*_ which crystallize in the disordered structure (*Fm*3̄*m*) and display a high capacity of ∼300 mA h g^−1^.^8^ Li_1.2_Ni_1/3_Ti_1/3_Mo_2/15_O_2_, another disordered compound, delivers a discharge capacity of ∼250 mA h g^−1^.^9^ Similarly, Li_(1+*x*)_Ti_2*x*_Fe_(1−3*x*)_O_2_,^10^ Li_1.3_Nb_0.3_V_0.4_O_2_,^11^ Li_2+2*x*_Mn_1−*x*_Ti_1−*x*_O_4_,^12^ Li_2_FeV_*y*_Ti_1−*y*_O_4_ (ref. 13) and Li_1.3_Ta_0.3_Mn_0.4_O_2_ (ref. 14) have also been studied recently. There are also successful efforts to replace oxide ions with fluoride ions to develop fluoride based disordered Li ion materials.^15–17^ Such recent progress shows that disordered oxides offer many possibilities for application to next generation LIBs.

In order to understand the stability/metastability of Li rich disordered materials compared to ordered layered cathode materials and the effect of excess lithium on the stability, we carried out high temperature oxide melt solution calorimetry on 
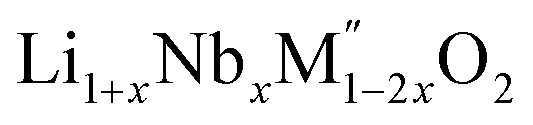
 (M′′ = Mn^3+^ and Fe^3+^ and *x* = 0.20, 0.30 and 0.40). We also synthesized Li_1+*x*_Ta_*x*_Fe_1−2*x*_O_2_ (*x* = 0.20, 0.30 and 0.40), new Ta containing Li excess disordered cathode materials, by a rapid quenching method and performed high temperature calorimetry to understand the role of the d^0^ pentavalent cations (Nb and Ta) on energetics. The results give understanding of the formation and stability of Li rich disordered cathode materials.

## Experimental methods

### Synthesis procedures and structural characterization


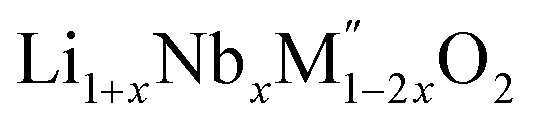
 (M′′ = Mn^3+^ and Fe^3+^, *x* = 0.2, 0.3, 0.4) samples were prepared by conventional solid state method by following the procedure reported earlier.^8^ The stoichiometric amounts of Li_2_CO_3_, Nb_2_O_5_, MnC_2_O_4_·2H_2_O and Fe_2_O_3_ were mixed and 0.1 mol of excess Li_2_CO_3_ was added to compensate the Li vaporization loss at high temperature. The mixture was heated between 950 and 975 °C to form the pure compounds. Li_1+*x*_Ta_*x*_Fe_1−2*x*_O_2_ (*x* = 0.20, 0.30 and 0.40) were formed by a rapid quenching method done in three steps. In the first step, stoichiometric amounts of Li_2_CO_3_, Ta_2_O_5_ and Fe_2_O_3_ with excess Li_2_CO_3_ were ground and heated in an alumina crucible at 810 °C to decompose the carbonate. Then the crucible with the sample was suspended in a vertical quenching tube furnace and heated to 1085 °C for 16 h. At the final step, the sample was quenched by dropping into liquid nitrogen. The quenching of the sample from high temperature maximizes the retention of a disordered high entropy state.

The samples were characterized by powder X-ray diffraction (PXRD) with a Bruker D8 (AXS) Advance diffractometer with Cu Kα radiation (40 kV, 40 mA) in the 2*θ* range of 10 to 80° with a 0.018 step size and a 6 s step time. The PXRD patterns were refined using the program GSAS-II.^18^ A ninth-order cosine Fourier polynomial for the background, zero, LP factor, scale, pseudo-Voigt profile function (*U*, *V*, *W*, and *X*), lattice parameters, atomic parameters, and *U*_iso_ (total 23 parameters) were used in refinement. The thermal parameters were constrained to be the same for atoms that occupied the same site (Li, Nb/Ta and Fe/Mn in all the compounds). The crystal structures are illustrated using VESTA software.^19^

### High temperature oxide melt solution calorimetry

High temperature oxide melt solution calorimetry was performed employing a custom build Tian–Calvet twin calorimeter.^20–23^ Around 5 mg of pelletized sample was dropped into molten sodium molybdate (3Na_2_O·4MoO_3_) solvent at 700 °C of 
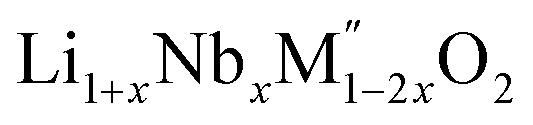
 (M′′ = Mn and Fe, *x* = 0.2, 0.3, 0.4) and at 800 °C for Li_1+*x*_Ta_*x*_Fe_1−2*x*_O_2_ (*x* = 0.2, 0.3, 0.4) to determine the drop solution enthalpies (Δ*H*_ds_) at the respective temperatures. The higher temperature used for tantalates ensured their rapid dissolution in the melt. The drop solution enthalpies of Li_1+*x*_Nb_*x*_Fe_1−2*x*_O_2_ are also measured at 800 °C in molten sodium molybdate (3Na_2_O·4MoO_3_) solvent to comparison with that of Li_1+*x*_Ta_*x*_Fe_1−2*x*_O_2_ compounds. The calorimetry glassware was flushed by oxygen gas at a flow rate of 30 ml min^−1^ and the solvent was bubbled with the same gas at 5 ml min^−1^ throughout each measurement. At least 8–10 experiments were done per sample and the results are reported as average values with error being two standard deviations of the mean. The calorimeter was calibrated using the heat content of 5 mg pellets of α-Al_2_O_3_. The details of the calorimeter and procedures have been described previously.^20–23^

## Results and discussion

### Synthesis and structure

**Fig. 1:**
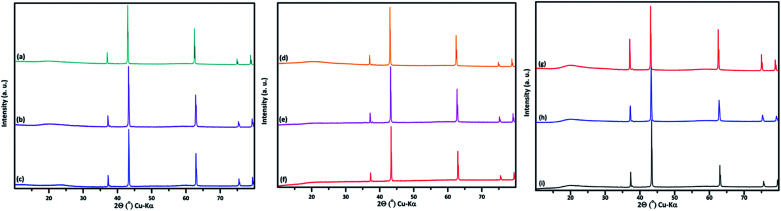
PXRD patterns of Li_1+*x*_Nb_*x*_Mn_1−2*x*_O_2_ (a) *x* = 0.2, (b) *x* = 0.3, (c) *x* = 0.4, Li_1+*x*_Nb_*x*_Fe_1−2*x*_O_2_ (d) *x* = 0.2, (e) *x* = 0.3, (f) *x* = 0.4 and Li_1+*x*_Ta_*x*_Fe_1−2*x*_O_2_ (g) *x* = 0.2, (h) *x* = 0.3, (i) *x* = 0.4. The small peak around 2*θ* = 38° is due to the instrument holder.

**Fig. 2:**
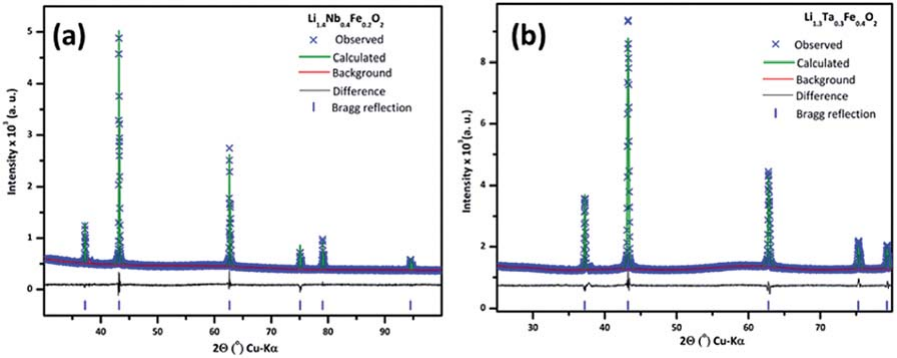
PXRD Rietveld refinement profile of (a) Li_1.4_Nb_0.4_Fe_0.2_O_2_ and (b) Li_1.3_Ta_0.3_Fe_0.4_O_2_. Observed (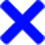), calculated (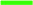) and difference (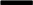) profiles are shown. The vertical blue bars (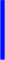) at the bottom indicate Bragg reflections corresponding to respective space group. The small peak around 2*θ* = 38° is due to the instrument holder.

**Fig. 3:**
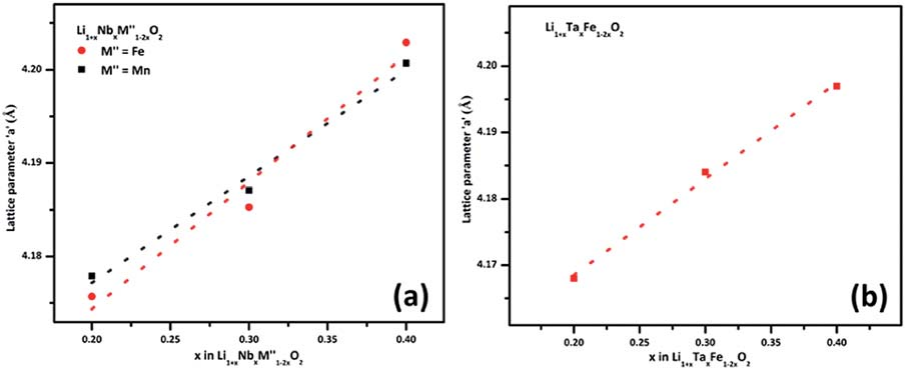
Unit cell parameter, ‘*a*’ *vs.* excess Li content, ‘*x*’, per formula unit of 
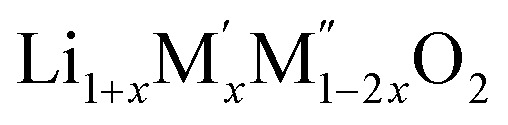
 (*x* = 0.20, 0.30 and 0.40) (a) M′ = Nb^5+^, M′′ = Mn^3+^ (black) and Fe^3+^ (red) and (b) M′ = Ta^5+^ and M′′ = Fe^3+^ (red).

PXRD shows that all the compounds in the series 
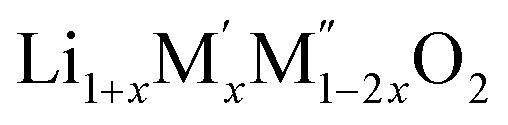
 (M′ = Nb and Ta, M′′ = Mn and Fe and *x* = 0.2, 0.3, 0.4) formed single phases in disordered rocksalt structure similar to (Li_3_NbO_4_)_*x*_–(LiM^3+^O_2_)_1−*x*_ (ref. 8) and Li_1.3_Ta_0.3_Mn_0.4_O_2_ (ref. 14) (Fig. 1). The Rietveld refinement was carried out on series 
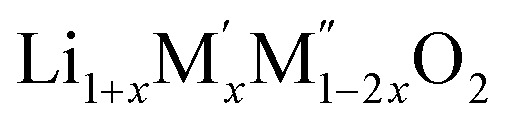
 (M′ = Nb and Ta, M′′ = Mn and Fe and *x* = 0.2, 0.3, 0.4) using the disordered structural model with the cubic *Fm*3̄*m* space group reported in the literature based on synchrotron X-ray and neutron diffraction studies of Li_1.3_Nb_0.3_Mn_0.4_O_2_ (ref. 24) and Li_1.3_Ta_0.3_Mn_0.4_O_2_ single crystals.^14^ In the crystal structure, 4b sites are occupies by oxygen atoms forming a cubic close packed structure and Li, M′, and M′′ atoms are randomly distributed in 4a sites. Rietveld refinement profiles of representative members, Li_1.4_Nb_0.4_Fe_0.2_O_2_ and Li_1.3_Ta_0.4_Fe_0.3_O_2_, are shown in Fig. 2. The refinement profiles of all other compounds are given in Fig. S1–S3 of ESI.† A summary of crystal structural data for all the compounds is given in Tables 1 and 2. Fig. 3 shows the increase of lattice parameter ‘*a*’ with excess lithium content, ‘*x*’, in 
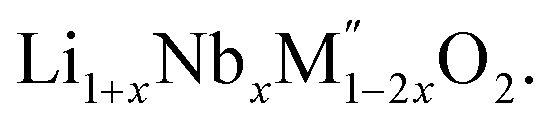
 This increase mirrors the increase in the average ionic radius of the cations in the octahedral coordination (ionic radius of Li^+^ – 0.76 Å, Mn^3+^/Fe^3+^ – 0.645 Å, Nb^5+/^Ta^5+^ – 0.64 Å).^25^

**Table 1:** Crystallographic data obtained from PXRD data for 
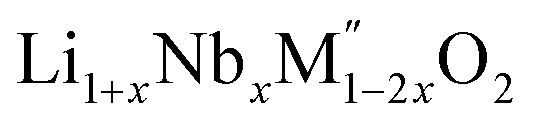
 compounds

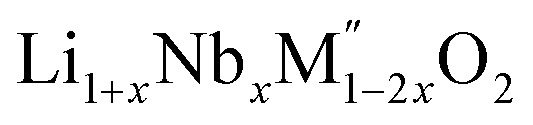		M′′ = Mn^3+^			M′′ = Fe^3+^		
		*x* = 0.2	*x* = 0.3	*x* = 0.4	*x* = 0.2	*x* = 0.3	*x* = 0.4
Space group		*Fm*3̄*m*					
Wyckoff position 4a (0,0,0)	‘Li’ occupancy	0.61(1)	0.65(1)	0.70(1)	0.60(1)	0.65(1)	0.69(1)
	‘Nb’ occupancy	0.11(1)	0.15(1)	0.20(1)	0.10(1)	0.15(1)	0.20(1)
	‘M’ occupancy	0.28(1)	0.20(1)	0.10(1)	0.30(1)	0.20(1)	0.11(1)
Wyckoff position 4b (0,0.5,0.5)	‘O’ occupancy	1.00	1.00	1.00	1.00	1.00	1.00
*U*_iso_ (Å)	‘Li/Nb/M’ site	0.002(1)	0.002(1)	0.007(1)	0.009(1)	0.004(1)	0.004(1)
	‘O’ site	0.044(1)	0.016(1)	0.029(1)	0.013(1)	0.012(1)	0.011(1)
Unit cell parameter ‘*a*’ (Å)		4.1779(1)	4.1871(1)	4.2007(1)	4.1757(1)	4.1853(1)	4.2029(1)
*R*_wp_ (%)		2.75	3.08	4.09	1.48	1.90	2.41
GOF		1.90	2.01	2.22	1.42	1.59	1.69

**Table 2:** Crystallographic data obtained from PXRD data for Li_1+*x*_Ta_*x*_Fe_1−2*x*_O_2_ compounds

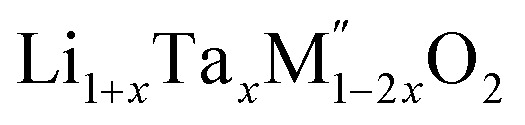		M′′ = Fe^3+^		
		*x* = 0.2	*x* = 0.3	*x* = 0.4
Space group		*Fm*3̄*m*		
Wyckoff position 4a (0,0,0)	‘Li’ occupancy	0.61(1)	0.65(1)	0.70(1)
	‘Ta’ occupancy	0.11(1)	0.15(1)	0.20(1)
	‘Fe’ occupancy	0.28(1)	0.20(1)	0.10(1)
Wyckoff position 4b (0,0.5,0.5)	‘O’ occupancy	1.00	1.00	1.00
*U*_iso_ (Å)	‘Li/Ta/Fe’ site	0.002(1)	0.002(1)	0.007(1)
	‘O’ site	0.044(1)	0.016(1)	0.029(1)
Unit cell parameter ‘*a*’ (Å)		4.1779(1)	4.1871(1)	4.2007(1)
*R*_wp_ (%)		1.97	1.92	2.24
GOF		2.27	2.25	2.09

### High temperature oxide melt solution calorimetry

The enthalpies of drop solution and enthalpies of formation of 
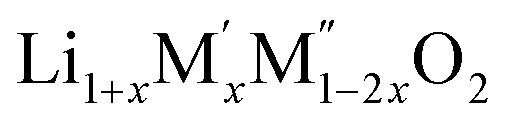
 (M′ = Nb and Ta, M′′ = Mn and Fe and *x* = 0.2, 0.3, 0.4) compounds are given in Table 3. The enthalpy of drop solution of lithium oxide (Li_2_O), which is hygroscopic and corrosive, was calculated from the drop solution enthalpy of lithium carbonate (Li_2_CO_3_) at 700 °C using the thermochemical cycle 1 in the Table 4. The enthalpies of drop solution of other binary oxides are taken from the literature.^20,26,27^ The enthalpies of formation from oxides at 25 °C 
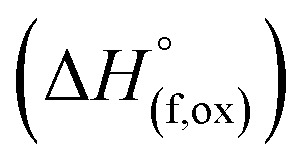
 for 
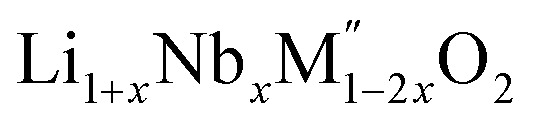
 compounds (Table 3) are calculated using the thermochemical cycle 2 in Table 4. The enthalpies of formation from oxides 
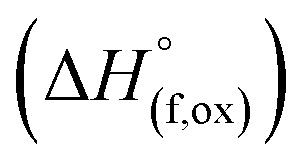
 for Li_1+*x*_Ta_*x*_Fe_1−2*x*_O_2_ are calculated using thermochemical cycle 3 in Table 4.

**Table 3:** Drop solution enthalpies in molten sodium molybdate solvent at 700 °C and at 800 °C and enthalpies of formation from oxides at 25 °C 
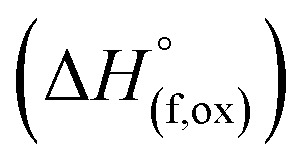
 of 
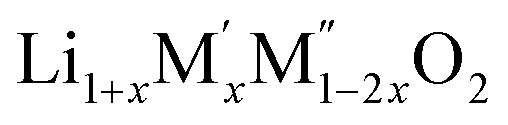
 (M′ = Nb^5+^ and Ta^5+^, M′′ = Mn^3+^ and Fe^3+^)

*x* in 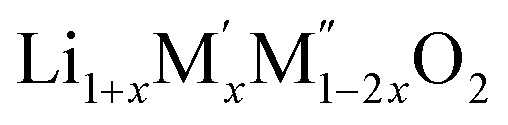	Δ*H*_ds_ (kJ mol^−1^)	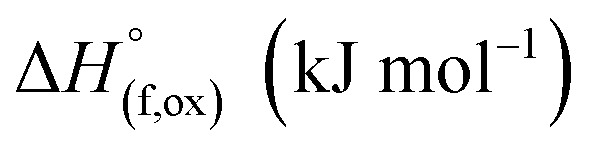
**Li**_**1+*x***_ **Nb**_***x***_ **Fe**_**1−2*x***_ **O**_**2**_ **measured at 700 °C**		
0.2	45.63 ± 0.53 (8)^a^	−61.72 ± 1.61
0.3	44.84 ± 0.51 (8)	−70.31 ± 1.72
0.4	41.97 ± 0.36 (8)	−76.82 ± 1.82

**Li**_**1+*x***_ **Nb**_***x***_ **Mn**_**1−2*x***_ **O**_**2**_ **measured at 700 °C**		
0.2	68.71 ± 0.60 (8)	−67.08 ± 1.65
0.3	61.34 ± 0.53 (8)	−74.89 ± 1.74
0.4	51.09 ± 0.50 (8)	−80.04 ± 1.85

**Li**_**1+*x***_ **Nb**_***x***_ **Fe**_**1−2*x***_ **O**_**2**_ **measured at 800 °C**		
0.2	66.06 ± 1.40 (8)	−65.57 ± 2.51
0.3	65.33 ± 0.32 (8)	−73.96 ± 2.24
0.4	63.71 ± 0.50 (8)	−81.46 ± 2.37

**Li**_**1+*x***_ **Ta**_***x***_ **Fe**_**1−2*x***_ **O**_**2**_ **measured at 800 °C**		
0.2	46.56 ± 0.80 (8)	−47.70 ± 2.23
0.3	49.14 ± 0.76 (8)	−60.22 ± 2.31
0.4	52.29 ± 0.91 (8)	−73.30 ± 2.48

a Number of drops given in parentheses.

**Table 4:** Thermochemical cycles employed to calculate the drop solution enthalpy of lithium carbonate (Li_2_CO_3_) at 700 °C (cycle 1), enthalpies of formation from oxides at 25 °C 
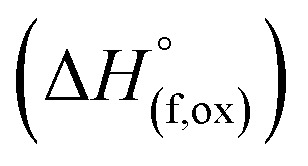
 for 
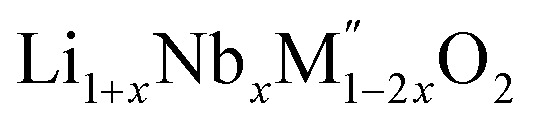
 (M′′ = Mn and Fe) (cycle 2) and enthalpies of formation from oxides 
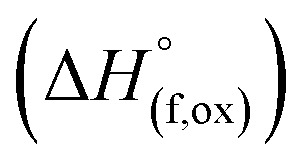
 for 
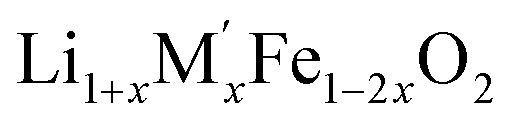
 (M′ = Nb^5+^ and Ta^5+^) (cycle 3)

Reaction		Δ*H* (kJ mol^−1^)
**Cycle 1: Δ*H***_**ds**_ **of Li**_**2**_ **O from Δ*H***_**ds**_ **of Li**_**2**_ **CO**_**3**_ **measured at 700 °C**		
Li_2_CO_3(s,25 °C)_ → Li_2_O_(sln,700 °C)_ + CO_2(g,700 °C)_	[1] Δ*H*_1_	161.28 ± 1.75
Li_2_O_(s,25 °C)_ + CO_2(g,25 °C)_ → Li_2_CO_3(g,25 °C)_	[2] Δ*H*_2_	−223.79 ± 2.11^a^
CO_2(g,25 °C)_ → CO_2(g,700 °C)_	[3] 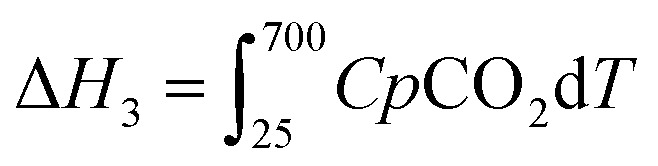	31.94^a^
Li_2_O_(s,25 °C)_ → Li_2_O_(sln,700 °C)_	[4] Δ*H*_4_	−94.46 ± 2.74
Δ*H*_4_ = Δ*H*_1_ + Δ*H*_2_ − Δ*H*_3_		

**Cycle 2:** 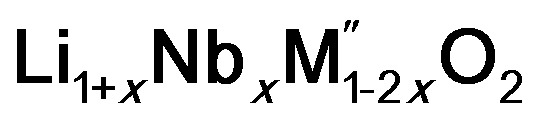 **(M′′ = Mn and Fe), Δ*H***_**ds**_ **measured at 700 °C**		
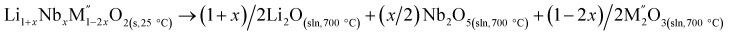	[5] Δ*H*_ds_ − Li_1+*x*_Nb_*x*_M_1−2*x*_O_2_	Table 3
Li_2_O_(s,25 °C)_ → Li_2_O_(sln,700 °C)_	[4] Δ*H*_ds_ − Li_2_O	−94.46 ± 2.74
Nb_2_O_5(s,25 °C)_ → Nb_2_O_5(sln,700 °C)_	[6] Δ*H*_ds_ − Nb_2_O_5_	93.97 ± 0.1.60^b^
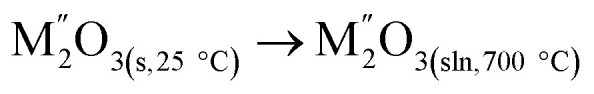	[7] 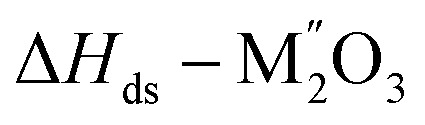	
M′′ = Fe, Fe_2_O_3(s,25 °C)_ → Fe_2_O_3(sln,700 °C)_	Δ*H*_ds_ − Fe_2_O_3_	95.63 ± 0.50^b^
M′′ = Mn, Mn_2_O_3(s,25 °C)_ → Mn_2_O_3(sln,700 °C)_	Δ*H*_ds_ − Mn_2_O_3_	154.70 ± 1.00^b^
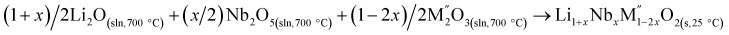	[8] 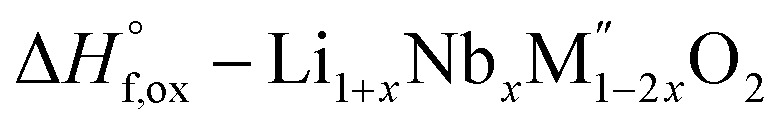	Table 3
Δ*H*[8] = −Δ*H*[5] + (1 + *x*)/2Δ*H*[4] + (*x*/2)Δ*H*[6] + (1 − 2*x*)/2Δ*H*[7]		

**Cycle 3:** 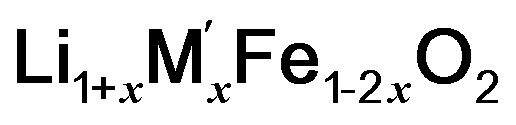 **(M′ = Nb and Ta) Δ*H***_**ds**_ **measured at 800 °C**		
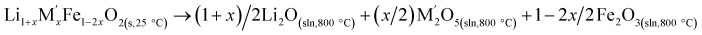	[9] 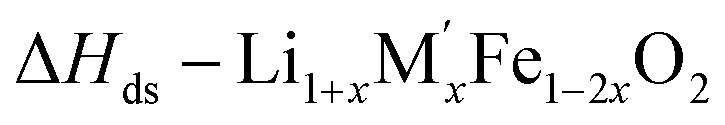	Table 3
Li_2_O_(s,25 °C)_ → Li_2_O_(sln,800 °C)_	[10] Δ*H*_ds_ − Li_2_O	−78.32 ± 3.28^c^
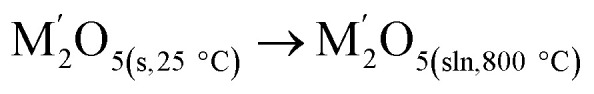	[11] 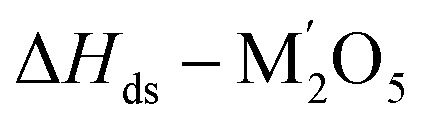	
M′ = Nb, Nb_2_O_5(s,25 °C)_ → Nb_2_O_5(sln,800 °C)_	Δ*H*_ds_ − Nb_2_O_5_	127.50 ± 0.80^b^
M′ = Ta, Ta_2_O_5(s,25 °C)_ → Ta_2_O_5(sln,800 °C)_	Δ*H*_ds_ − Ta_2_O_5_	111.18 ± 1.00^d^
Fe_2_O_3(s,25 °C)_ → Fe_2_O_3(sln,800 °C)_	[12] Δ*H*_ds_ − Fe_2_O_3_	115.78 ± 2.20^e^
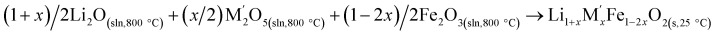	[13] 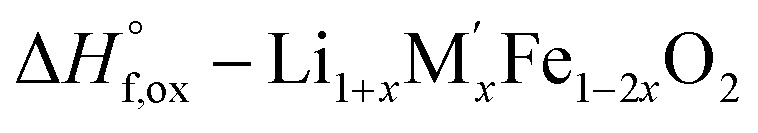	Table 3
Δ*H*[13] = −Δ*H*[9] + (1 + *x*)/2Δ*H*[10] + (*x*/2)Δ*H*[11] + (1 −2*x*)/2Δ*H*[12]		

a Taken from ref. 26 and 27.

b Taken from ref. 20.

c The enthalpy of drop solution of Li_2_O at 800 °C is calculated from the enthalpy of drop solution of Li_2_CO_3_ at 800 °C. The experiments were done by M. Abramchuk and A. Navrotsky [results unpublished].

d The enthalpy of drop solution of Ta_2_O_5_ at 800 °C used here is measured by S. Hayun, S. J. McCormack, K. I. Lilova and A. Navrotsky [results unpublished].

e The enthalpy of drop solution of Fe_2_O_3_ at 800 °C used here is measured by S. Hayun and A. Navrotsky [results unpublished].

## Discussion

**Fig. 4:**
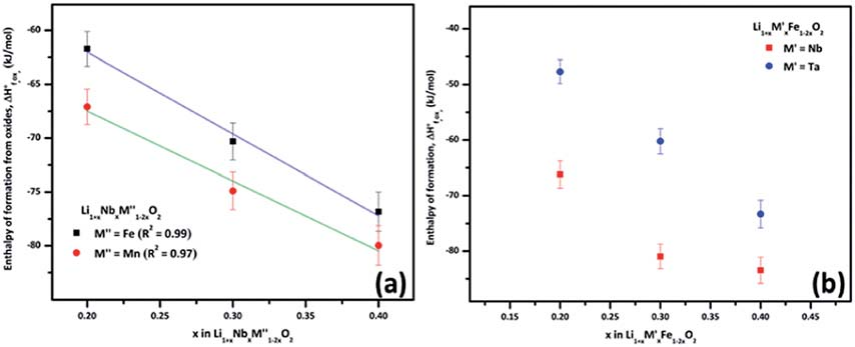
(a) Enthalpy of formation from oxides 
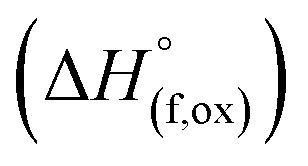
*vs.* excess Li content ‘*x*’ per formula unit of 
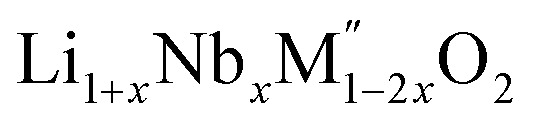
 (M′′ = Mn^3+^ and Fe^3+^ and *x* = 0.20, 0.30 and 0.40) and (b) enthalpy of formation from oxides 
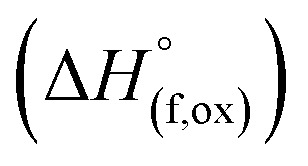
*vs.* excess Li content ‘*x*’ per formula unit of 
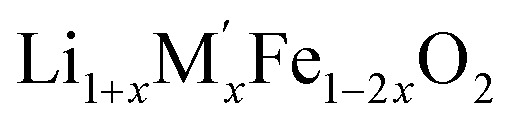
 (M′ = Nb^5+^ and Ta^5+^ and *x* = 0.20, 0.30 and 0.40).

A plot of formation enthalpies from oxides *vs.* excess Li content ‘*x*’ per formula unit of 
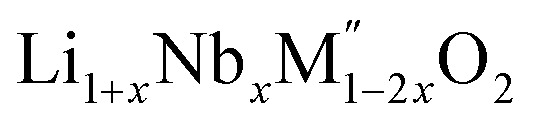
 (Fig. 4(a)) shows that the enthalpies of formation of 
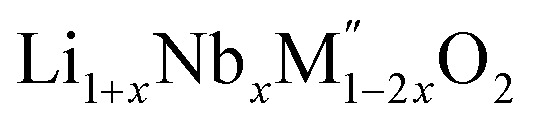
 oxides become more exothermic as the excess Li per formula unit (*x*) increases for both M = Mn and Fe. More exothermic formation enthalpies with increasing Li content in the ordered Li_1−*x*_CoO_2_ and Li_1+*x*_Mn_2−*x*_O_4_ compounds have been reported previously.^28–30^ The result suggests that the compounds become thermodynamically more stable when excess Li ions are introduced in the structure. The increase in the stability with higher Li content is most likely due to acid–base reaction between more basic Li_2_O bonds and more acidic transition metal oxides. Further, a comparison of formation enthalpies of Li_1+*x*_Nb_*x*_Mn_1−2*x*_O_2_ and Li_1+*x*_Nb_*x*_Fe_1−2*x*_O_2_ (Fig. 4(a) and Table 3) displays that Mn and Fe analogues have almost equal formation enthalpies with in the experimental error. A comparison of formation enthalpies of Li_1+*x*_Nb_*x*_Fe_1−2*x*_O_2_ and Li_1+*x*_Ta_*x*_Fe_1−2*x*_O_2_ (Fig. 4(b) and Table 3) reveals that niobium compounds have more exothermic formation enthalpies than the tantalum compounds. The higher exothermic formation enthalpies of niobium compounds could be result of difference in the acidity between Nb_2_O_5_ and Ta_2_O_5_ along with the kinetically controlled factors affecting the formation of 
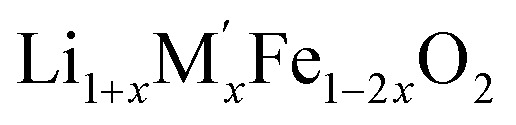
 (M′ = Nb^5+^ and Ta^5+^) compounds.

### Configurational entropy of disordered 
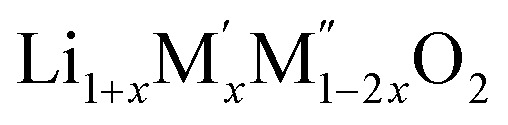
 (M′ = Nb^5+^ and Ta^5+^, M′′ = Mn^3+^ and Fe^3+^)

The higher entropy stabilizes the disordered Li cathode materials at high temperature. The entropy term arises from two sources: (1) lattice (vibrational and magnetic) and (2) configurational. In the disordered Li materials, the configurational entropy due to the cation disordering contributes more to the Gibbs free energy than the vibrational entropy term at higher temperature. To quantify the contribution of the configurational entropy to the stability, the configurational entropy of disordered Li materials is calculated by following the method of Navrotsky and Kleppa^31^ and O'Neill and Navrotsky.^32–34^

Short-range cation ordering has been reported in disordered Li ion materials.^14,35,36^ However, the extent of short range order has not been clearly given. Hence, the random model reported based on the synchrotron XRD and neutron diffraction studies of and Li_1.3_Nb_0.3_Mn_0.4_O_2_ single crystals^24^ has been used for the configurational entropy calculations in the present work considering complete disorder. The effect of short range ordering on the configurational entropy is discussed below. The configurational entropy calculation for one of the members, Li_1.3_Nb_0.3_Fe_0.4_O_2_, is explained in detail. If Li_1.3_Nb_0.3_Fe_0.4_O_2_ is completely ordered in layered rock salt structure, 1.0 Li ions occupy distinct crystallographic positions in one layer (slab layer) and 0.3 Li, 0.3 Nb, 0.3 Fe ions occupy distinct crystallographic positions in alternate layer (interslab layer) as in case of Li_2_MO_3_ (Li_1.33_M_0.67_O_2_) where M = Ti, Ru and Sn.^37–39^ The molecular formula of the ordered structure can be written as [Li]_slab_[Li_0.3_Nb_0.3_Fe_0.4_]_interslab_O_2_. The process of disordering is expected to proceed through two steps (Fig. S4†). In the first step, there will be a random distribution of cations (Li, Nb, and Fe) in the interslab layer, [Li_0.3_Nb_0.3_Fe_0.4_]_interslab_ layer, followed by random mixing of cations between the slab, [Li]_slab_, and interslab, [Li_0.3_Nb_0.3_Fe_0.4_]_interslab_, layers in the second step. These two factors contribute to the configurational entropy (*S*_c_), one given by random mixing of cations in the interslab layers (*S*_c,interslab_) and the other given by random mixing of cations between the slab and interslab layers (*S*_c,interslab–slab_). The configurational entropy arising from by random mixing of cations interslab layers, [Li_0.3_Nb_0.3_Fe_0.4_]_interslab_O, (*S*_c,interslab_) is calculated by*S*_c,interslab_ = −*R*[0.3 ln 0.3 + 0.3 ln 0.3 + 0.4 ln 0.4] = 9.062 J K^−1^ mol^−1^

In the completely disordered state, 0.35 Li ions from slab layer occupy the cations sites in the interslab layer and 0.15 Nb and 0.2 Fe ions from interslab layer occupy the cations sites in the slab layer to yield the molecular formula [Li_0.65_Nb_0.15_Fe_0.2_]_slab_[Li_0.65_Nb_0.15_Fe_0.2_]_interslab_O_2_. The configurational entropy arising from by random mixing of cations between the slab and interslab layers (*S*_c,interslab–slab_) is calculated by*S*_c,interslab–slab_ = −*R*[0.35 ln 0.35 + 0.15 ln 0.15 + 0.2 ln 0.2]*S*_c,interslab–slab_ = 8.098 J K^−1^ mol^−1^

The sum of these two configurational entropies gives the total configurational entropy of the system.*S*_c_ = *S*_c,interslab_ + *S*_c,interslab–slab_ = 17.160 J K^−1^ mol^−1^

Similarly, the configurational entropies for all the compounds are calculated and given in Table S1.† Since the configurational entropy of the fully ordered state is zero, the change in the configurational entropy due to disorder, Δ*S*_c_, will be as same as the configurational entropy, *S*_c_, of the disordered state. The contribution of *T*Δ*S*_c_ term to the Gibbs free energy at room temperature for all compounds and given in Table S1.† This stabilization due to the configurational entropy would be decreased if there were short-range ordered domains.

Syntheses of ordered polymorphs of all the compounds have been attempted by slow cooling but without success. Only the mixture of ordered end members, Li_3_M′O_4_ (M′ = Nb and Ta) and LiM′′O_2_ (M′′ = Fe and Mn), were formed by slow cooling. The small enthalpy difference between ordered and disordered structures can be illustrated by taking the case of LiFeO_2_ which has both ordered (γ) and disordered (α) polymorphs. The enthalpies of formation of ordered (γ) and disordered (α) LiFeO_2_ have been determined to be −46.95 ± 1.34 kJ mol^−1^ and −37.74 ± 1.28 kJ mol^−1^.^26^

The enthalpy of the order-disorder transition, Δ*H*_trans_, is 9.21 ± 1.85 kJ mol^−1^. The Gibbs free energy of transition, Δ*G*_trans_, is given by Δ*G*_trans_ = Δ*H*_trans_ − *T*_trans_Δ*S*_trans_. In the same work, the order-disorder transition temperature, *T*_trans_, is given as 475 °C (748 K). At equilibrium, Δ*G*_trans_ = 0. Thus, Δ*S*_trans_ = Δ*H*_trans_/*T*_trans_ and is 12.34 J K^−1^ mol^−1^. Since 0.5Li and 0.5Fe cations are distributed at 4a site in α-LiFeO_2_,^40^ the configurational entropy of α-LiFeO_2_ is given by *S*_c,interslab_ = −*R*[0.5 ln 0.5 + 0.5 ln 0.5] = 5.82 J K^−1^ mol^−1^. Hence, for α-LiFeO_2_, the contribution of configurational entropy to Δ*S*_trans_ is 5.82 J K^−1^ mol^−1^ with 6.52 J K^−1^ mol^−1^ from lattice entropy an almost equal contribution. This is in case of α-LiFeO_2_ which has disorder only between cations in the slab and interslab layers.

In the disordered Li cathode materials, the contribution of the configurational entropy is expected to be larger due to the substitution and random mixing of cations both within the interslab and between slab and interslab layers. The contribution of the configurational entropy to the Gibbs free energy is relatively small at room temperature (Table S1†). However at the temperature of synthesis (1085 °C/1358 K), the −*T*Δ*S* term (where *T* is temperature *T* = 1358 K and Δ*S* is entropy change, Δ*S* = 16.00 ± 0.55 J K^−1^ mol^−1^ (average entropy value from Table S1†)) is more significant, contributing −22.00 ± 0.55 kJ mol^−1^ to the free energy and thus stabilizing the disordered phase.

The aim of calculating configurational entropy is to quantify the contribution of the entropy term for the stabilization of the disordered compounds at the high temperature at which they are synthesized. Short range ordering at room temperature has been reported in Li_1.3_Nb_0.3_Mn_0.4_O_2_ and Li_1.3_Ta_0.3_Mn_0.4_O_2_ single crystals based on density functional theory calculations.^14,36^ In the calculations, cluster models with different cation ordering schemes have been used. In all the ordering schemes used (ESI of ref. 14 and 36), only 25% of the cations have been fixed in a site leaving 75% to be distributed randomly. The most stable cluster model has been found to be 
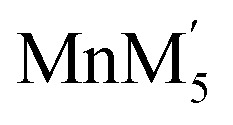
 (M′ = Nb and Ta) clusters with a random distribution of one Mn and five M′ cations at the fixed site. This randomness at the fixed site would further reduce the effect of short range ordering on the configurational entropy. As the temperature increases, short range order will diminish leading to a random distribution of cations which will increase the configurational entropy. The cluster based calculations have been carried out to understand how the short range ordering affects the Li ion mobility at room temperature in disordered Li materials. As the present study focuses on the stabilization of disordered structure by configurational entropy at high temperature at which synthesis are done, the effect of short range order will have at most a minor effect on the configurational entropy term to the stabilization. Jones *et al.* concluded that the correlation length of short range order in disordered Li_1.25_Nb_0.25_Mn_0.5_O_2_ depends on the post synthesis cooling rate, with rapid cooling leading to a shorter correlation length as expected on thermodynamic grounds.^41^ The present work quantifies the contribution of configurational entropy to the entropy term that stabilizes the disordered structure at high temperature with other contribution being given by lattice entropy (vibrational and magnetic). Thus, heating to higher temperature increases the randomness of the atoms leading to complete disorder and the rapid quenching from higher temperature preserves the disorder. This variation may suggest that tailoring the cooling regimen might decrease short range ordering and lead to higher Li ion mobility to deliver higher discharge capacities and energy densities.

## Conclusions


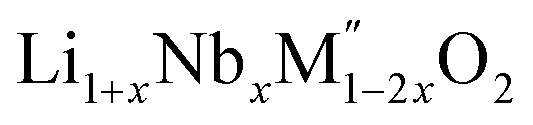
 (M′′ = Mn^3+^ and Fe^3+^ and *x* = 0.20, 0.30 and 0.40) and a new family of Ta containing Li excess disordered cathode materials, Li_1+*x*_Ta_*x*_Fe_1−2*x*_O_2_ (*x* = 0.20, 0.30 and 0.40) have been synthesized and high temperature oxide melt calorimetry experiments have been carried out. The enthalpies of formation of all the compounds show that increasing Li content per formula unit increases the stability irrespective of transition metal ions. The more exothermic enthalpies of formation for compounds with higher Li content are attributed to the reaction between more basic Li_2_O and more acidic transition metal oxides. The configurational entropy provides significant stabilization to the materials at high temperature.

## Conflicts of interest

There are no conflicts to declare.

## Supplementary Material

RA-010-C9RA09759G-s001
