# Supplementary material for: Thermochemistry of cation disordered Li ion battery cathode materials, [image] (M′ = Nb and Ta, M′′ = Mn and Fe)
Source: RSC Adv. 2020 Feb 12;10(11):6540–6. doi: 10.1039/c9ra09759g (PMC9049730; doi:10.1039/c9ra09759g)
Supplement: RA-010-C9RA09759G-s001 [file RA-010-C9RA09759G-s001.pdf]

Electronic Supplementary Material (ESI) for RSC Advances.
This journal is © The Royal Society of Chemistry 2020

Electronic Supplementary Information (ESI) for

Thermochemistry of cation disordered Li ion battery cathode materials, $Li_{1+x}M'_xM''_{1-2x}O_2$ (M' = Nb and Ta, M'' = Mn and Fe)

Tamilarasan Subramani[a] and Alexandra Navrotsky[a,b]*

[a]Peter A. Rock Thermochemistry Laboratory and NEAT ORU

University of California Davis

Davis CA 95616

[b]School of Molecular Sciences and Center for Materials of the Universe

Arizona State University

Tempe AZ 85287 Email: alexnav@asu.edu

Figures

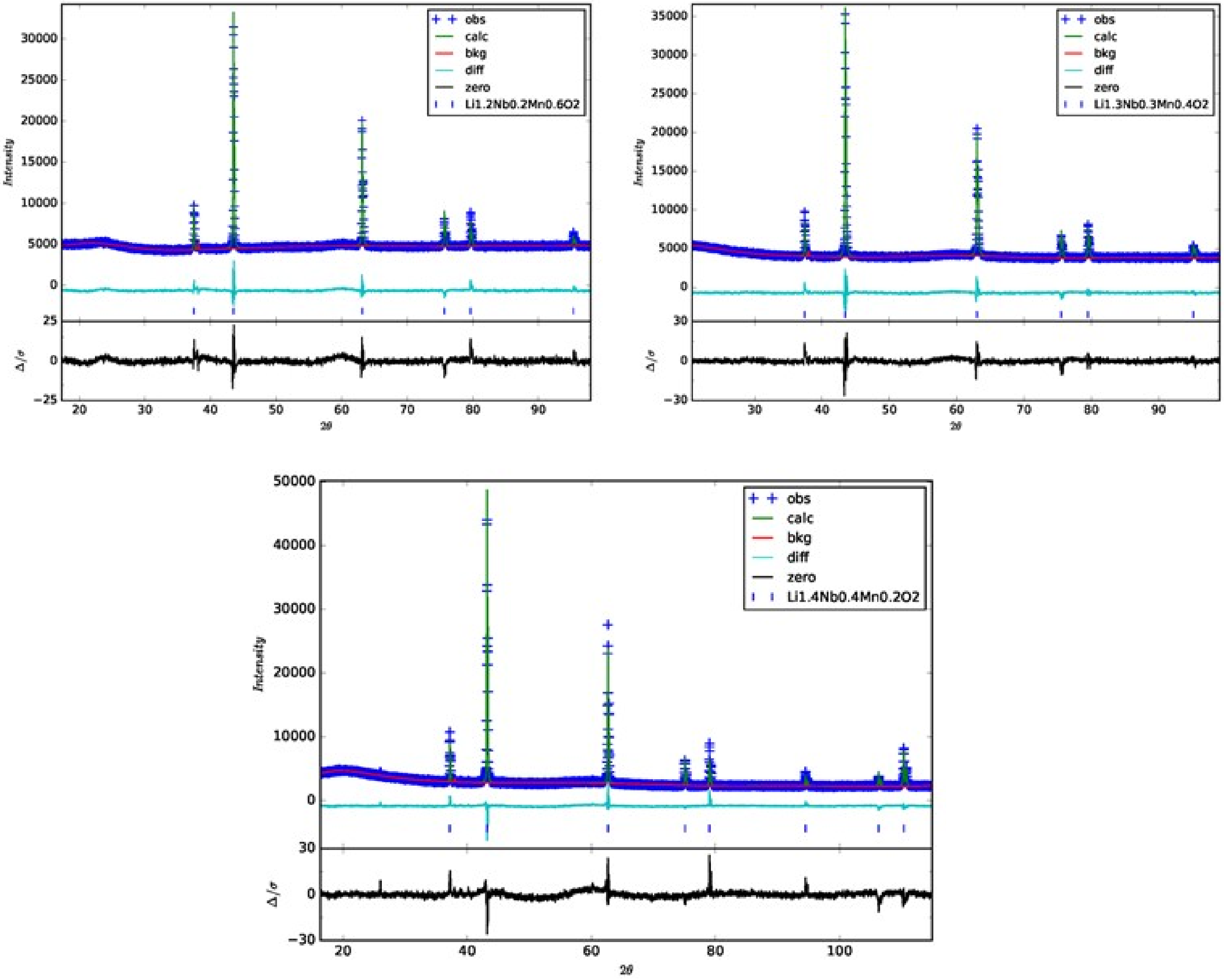

Fig. S1 PXRD Rietveld refinement profiles of $Li_{1-x}Nb_xMn_{1-2x}O_2$. Observed (+), calculated (–) and difference (–) profiles are shown. The vertical blue bars (|) at the bottom indicate Bragg reflections corresponding to respective space group. The small peak around 2θ = 38° is due to the instrument holder.

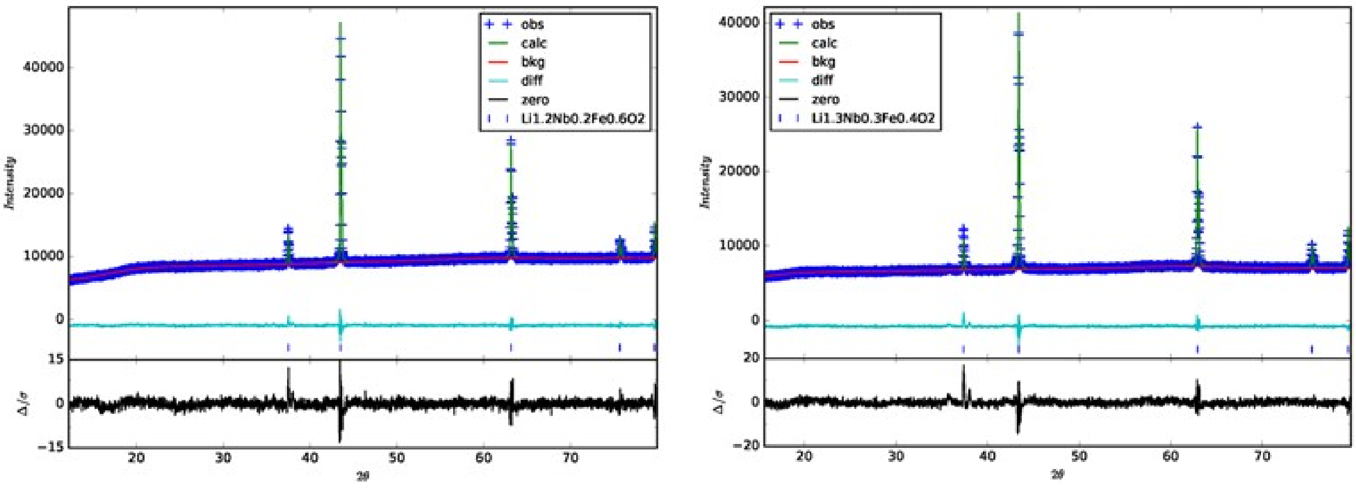

Fig. S2 PXRD Rietveld refinement profiles of $Li_{1-x}Nb_xFe_{1-2x}O_2$(x = 0.2 and 0.4). Observed (+), calculated (–) and difference (–) profiles are shown. The vertical blue bars (|) at the bottom indicate Bragg reflections corresponding to respective space group. The small peak around 2θ = 38° is due to the instrument holder.

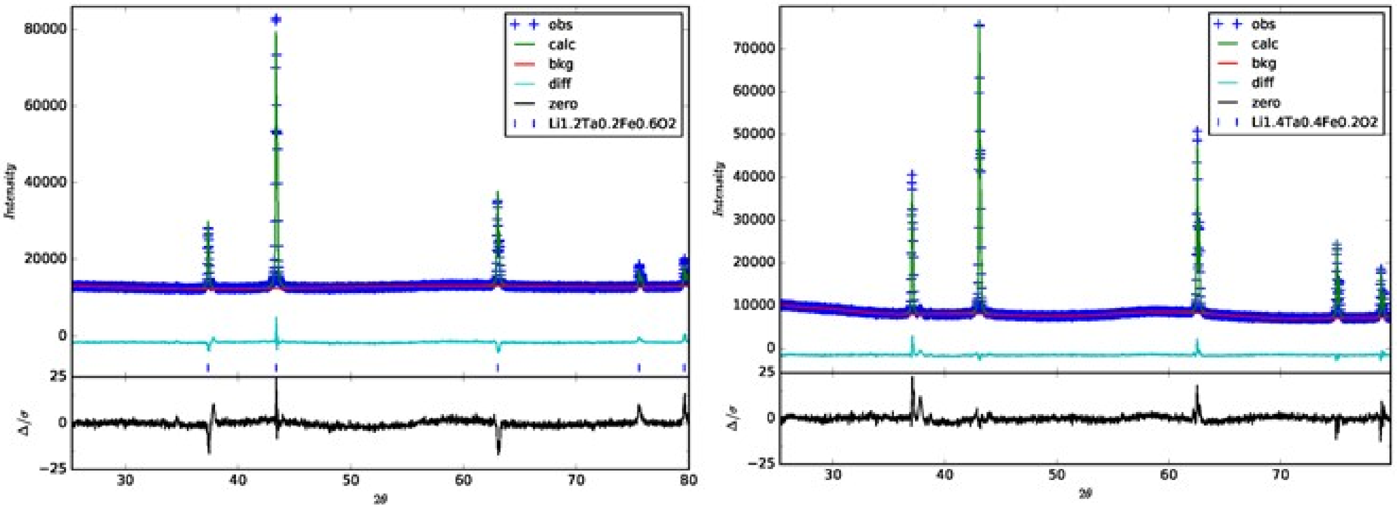

Fig. S3 PXRD Rietveld refinement profiles of $Li_{1-x}Ta_xFe_{1-2x}O_2$ (x = 0.2 and 0.4) Observed (+), calculated (–) and difference (–) profiles are shown. The vertical blue bars (|) at the bottom indicate Bragg reflections corresponding to respective space group. The small peak around 2θ = 38° is due to the instrument holder.

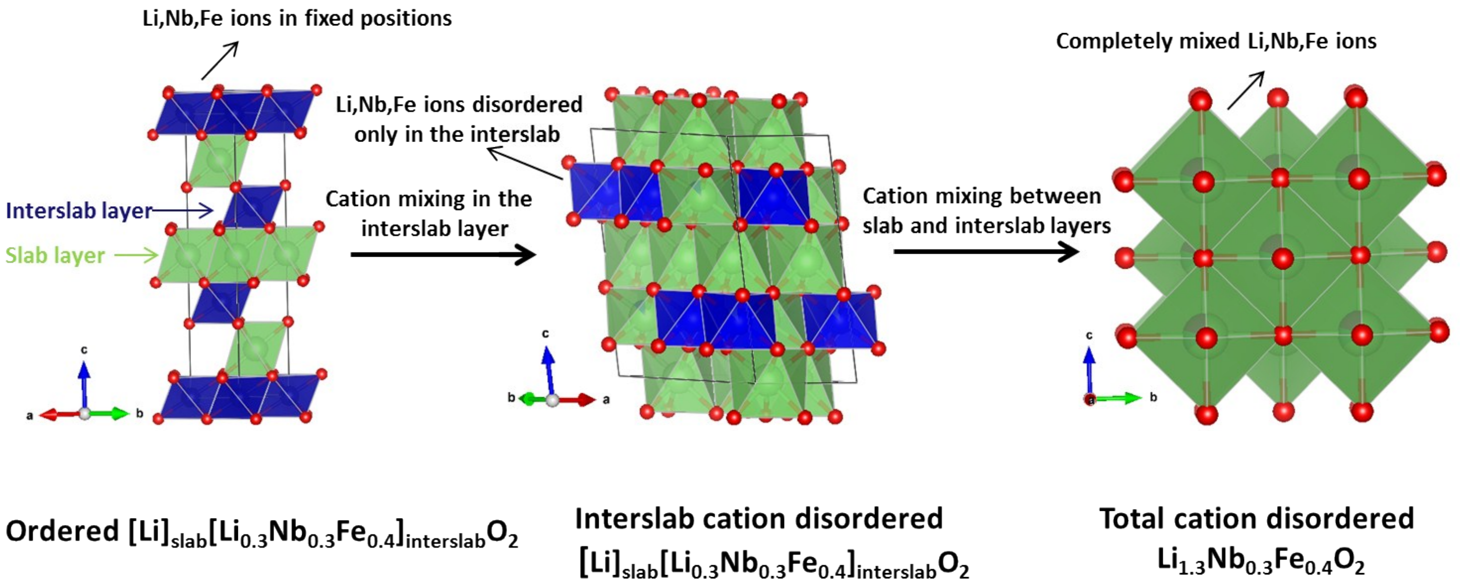

Fig. S4 The process of disordering: in the first step cations disorder only in the interslab layer and in the second step cations disorder between slab and interslab layers to give completely disordered $Li_{0.3}Nb_{0.3}Fe_{0.4}O_2$.

Table S1 Configurational entropy of mixing in the interslab, $S_{c,\ interslab}$, between interslab and slab, $S_{c,\ interslab-slab}$, total configurational entropy, S_c, and the term, $T\Delta S_c$ of all the compounds

Compound	$S_{c,\ interslab}$ ($JK^{-1}mol^{-1}$)	$S_{c,\ interslab-slab}$ ($JK^{-1}mol^{-1}$)	$S_{c,\ total}$ ($JK^{-1}mol^{-1}$)	$-T\Delta S_c$ (kJ/mol) at 298K
$Li_{1.2}Nb_{0.2}Mn_{0.6}O_2$	7.898	7.965	15.863	-4.73
$Li_{1.3}Nb_{0.3}Mn_{0.4}O_2$	9.062	8.098	17.160	-5.11
$Li_{1.4}Nb_{0.4}Mn_{0.2}O_2$	8.771	7.593	16.364	-4.88
$Li_{1.2}Nb_{0.2}Fe_{0.6}O_2$	7.898	7.965	15.863	-4.73
$Li_{1.3}Nb_{0.3}Fe_{0.4}O_2$	9.062	8.098	17.160	-5.11
$Li_{1.4}Nb_{0.4}Fe_{0.2}O_2$	8.771	7.593	16.364	-4.88
$Li_{1.2}Ta_{0.2}Fe_{0.6}O_2$	7.898	7.965	15.863	-4.73
$Li_{1.3}Ta_{0.3}Fe_{0.4}O_2$	9.062	8.098	17.160	-5.11
$Li_{1.4}Ta_{0.4}Fe_{0.2}O_2$	8.771	7.593	16.364	-4.88
